# RNA-sequencing analysis of the effect of luteolin on methamphetamine-induced hepatotoxicity in rats: a preliminary study

**DOI:** 10.7717/peerj.8529

**Published:** 2020-02-06

**Authors:** Dong Qu, Kaikai Zhang, Lijian Chen, Qi Wang, Huijun Wang

**Affiliations:** Department of Forensic Pathology, School of Forensic Medicine, Southern Medical University, Guangzhou, China

**Keywords:** Methamphetamine, Luteolin, Hepatotoxicity, Differentially expressed genes (DEGs), RNA-seq, qRT-PCR

## Abstract

In this study, RNA-sequencing (RNA-seq) was utilized to investigate the effects of luteolin on hepatotoxicity caused by methamphetamine (METH). The rats in METH group were administrated with METH (15 mg/kg, two times per day) via intraperitoneal (i.p.) injections for four consecutive days. The rats in luteolin + METH group were firstly administrated with luteolin (100 mg/kg, once a day) by oral gavage for 3 days before METH treatment. Lueolin attenuated the hepatotoxicity induced by METH via histopathological and biochemical analysis. The results of RNA-seq showed that luteolin could regulate 497 differentially expressed genes (DEGs), and the selected DEGs were mainly enriched in eight pathways, according to KEGG analysis. Furthermore, qRT-PCR was utilized to verify the results of RNA-seq. Six genes were selected as follows: liver enriched antimicrobial peptide 2 (Leap2), fatty acid synthase (Fasn), fatty acid binding protein 5 (Fabp5), patatin like phospholipase domain containing 3 (Pnpla3), myelin basic protein (Mbp) and calmodulin 3 (Calm3). Though because of the design flaws, the luteolin group has not been included, this study demonstrated that luteolin might exert hepato-protective effects from METH via modulation of oxidative phosphorylation, cytochrome P450 and certain signaling pathways.

## Introduction

Methamphetamine (METH) is an illegal psychostimulant that is abused all over the world (Courtney & Ray, 2014). For decades, research has reported that METH could cause neurotoxicity and a series of neurocognitive effects resulting in physical and psychological disorders in METH abusers (Jablonski, Williams & Vorhees, 2016; Kish et al., 2017; Lappin, Darke & Farrell, 2018; Loftis & Janowsky, 2014; Mursaleen & Stamford, 2016; Thanos et al., 2016). METH also causes multiple organ damage in abusers, and the liver is a significant target of METH damage. The main mechanisms of METH-induced hepatotoxicity are production of reactive metabolites, hyperthermia, increased neurotransmitter efflux, oxidation of biogenic amines, mitochondrial impairment, apoptosis and indirectly, genetic polymorphisms (Willson, 2019). Recently, our group focused on the hepatotoxicity caused by METH and also demonstrated that METH could cause liver injury in an experiment on rats (Wang et al., 2017a).

Luteolin (3, 4, 5, 7-tetrahydroxyflacone) is a type of natural flavonoid that exists in plants such as fruits (e.g., tamarind fruit, olive), herbs (e.g., Japanese honeysuckle, Florists Dendranthema, Scutellaria Linn), and vegetables (e.g., cauliflower, cabbage, green pepper) (Imran et al., 2019). Recently, luteolin has been reported to possess various biological advantages such as antitumor (Imran et al., 2019; Tuorkey, 2016), anti-inflammatory (Guo et al., 2017; Hytti et al., 2017; Kim et al., 2018), anti-oxidation (Ramos, Pereira-Wilson & Collins, 2010; Shi et al., 2015; Yu et al., 2015) and anti-allergy (Kritas et al., 2013) capabilities. The protective effect of luteolin on hepatotoxicity caused by tetrachloromethane (CCl_4_), acetaminophen, mercuric chloride and ethanol has been demonstrated in vivo and in vitro in recent studies (He et al., 2016; Liu et al., 2014; Tai et al., 2015; Zhang et al., 2017). Since hepatotoxicity due to different medicines and toxins can be attenuated by luteolin, we hypothesized that luteolin may have protective effects on hepatic injury due to METH. Thus, the following experiment was carried out to test the hypothesis that luteolin has protective effects upon METH-induced hepatotoxicity. As a result, we found that luteolin could obviously attenuate the hepatotoxicity caused by METH. However, the mechanism of effect of luteolin is still unknown.

Together, in this study, RNA-seq was performed to investigate the effect and mechanism of luteolin on hepatotoxicity caused by METH. A better understanding of the molecular mechanism involved in effect of luteolin on METH-induced hepatotoxicity might help identify some differentially expressed genes (DEGs) as targets for the development of anti-hepatotoxicity drugs.

## Materials and Methods

### Reagents

Methamphetamine was purchased from the National Institute for the Control of Pharmaceutical and Biological Products (Beijing, China, purity >99%). Luteolin was purchased form Push Bio-Technology Co., Ltd. (Chengdu, China, purity >96%).

### Experimental animals and treatments

Adult male Sprague Dawley (SD) rats weighing between 200 and 250 g, obtained from the Laboratory Animal Center of Southern Medical University, were randomly divided into three groups of six animals each and used for experiments. The rats were housed on a 12 h light-dark cycle (temperature 22 ± 2 °C) and habituated in tub cages for 7 days before use. All the animal procedures were conducted in accordance with Institutional Guidelines for the Care and Use of Laboratory Animals of Southern Medical University (SCKY (Guangdong) 44002100016156).

The acute hepatotoxicity animal model was induced by METH. The detailed procedure based on previous studies (Wang et al., 2017a; Xie et al., 2018b) was as follows: 15 mg/mL/kg, eight injections (i.p.) at 12 h intervals. Randomized grouping, three groups (six rats, each group; 18 rats in total): (1) control group: the rats were administered the solvents that were incorporated as experiment groups. They were administrated with 0.5% sodium dodecyl sulfate (SDS) by oral gavage for 3 days (once daily) and followed with normal saline injections for 4 days (i.p.). (2) METH group: the rats were administrated with 0.5% SDS for 3 days, then were given METH injections for 4 days (i.p.). (3) luteolin pre-treated group: luteolin (100 mg/kg, was administered by oral gavage for 3 days (once daily) (Tai et al., 2015)) before METH treatment. Luteolin was dissolved in 0.5% SDS water solution. After 12 h, all the rats were sacrificed under deep anesthesia with pentobarbital sodium (60 mg/kg i.p.). Blood samples and livers were taken from the rats immediately and placed into trace element free tubes. All the samples were kept at −80 °C.

### Animal welfare

Rats were housed in SPF-class animal laboratory with air-conditioning and 12 h dark-light cycle, ambient temperature at 23 °C (±2 °C), and relative humidity of 50%. Rats were feed food and tap water. The rats were acclimatized for at least one week. The procedures used were as humane as possible. All studies involving animals are reported in accordance with the ARRIVE and US NIH guidelines for reporting experiments involving animals.

### Histopathology

Liver tissue was removed from the rats and fixed by 10% formalin (v/v) solution for 24–48 h. The liver tissue was embedded in paraffin, and was then stained with hematoxylin and eosin (H&E). The pathological change area of the livers was observed under light microscope (DP-73; Olympus, Tokyo, Japan).

### Biochemical analysis

Blood samples were centrifuges at 800 g for 10 min at room temperature. Plasma was then used to evaluate the extent of hepatic injury by measuring serum levels of aspartate transaminase (AST) and alanine transaminases (ALT) via detected by Enzyme-Linked Immunosorbent Assay (CUSABIO Biotechnology, Wuhan, China ) (Wang et al., 2017a).

### RNA extraction and quality control

Trizol Reagent was used to extract total RNA from the liver tissue, and DNase I was used to remove gDNA from the total RNA. NanoDrop, 2100 Bioanalyser, agarose gel electrophoresis of RNA, and Qubi were utilized to analyze the quality and the quantity of the total RNA extracted from the liver tissue (Botling & Micke, 2011). The quality and quantity of the total RNA that was used to construct the transcriptome RNA-seq library met the following standards: OD260/280 = 1.8~2.2, OD260/230 ≥ 2.0, RIN ≥ 6.5, 28S:18S ≥ 1.0 and >10 μg.

### Library preparation and Illumina Hiseq xten Sequencing

A total of five μg of total RNA was pretreated for construction of the sequencing library according to the manufacturer’s instructions of the RNA preparation kit (Illumina, San Diego, CA, USA). Next the mRNA was isolated and enriched by oligo (dT) beads by combining with the polyA tail of mRNA. The isolated mRNA was fragmented into tiny fragments, and reverse transcripted to the complementary DNA (cDNA) in the form of double strands by using fragmentation buffer and a cDNA synthesis kit. Then the cDNA was pretreated according to the manufacturer’s instructions of the sequencing library construction. The cDNA of 200–300 bp in the cDNA pool was extracted by using agarose gel electrophoresis of DNA and amplified by PCR (Kumar et al., 2012). Lastly, the amplification product was sequenced by using the Illumina HiSeq xten (Major Biotechnology Company, Shanghai, China) according to the manufacturer’s instructions.

### Read mapping

In order to get clean reads, the raw reads should be filtered by the following standards: (1) remove the reads with adapters; (2) remove the unknown reads with a proportion of more than 10% in the raw reads; (3) remove the low-quality reads. After removing the mentioned three types of reads, the clean reads could be filtered. And then TopHat software was used to map the clean reads to reference the genome to get mapped reads (Trapnell, Pachter & Salzberg, 2009).

### Differential expression analysis and functional enrichment

In order to screen the DEGs in the three groups, the fragments per kilobase of exon per million mapped reads was utilized to calculate the relative quantity of each gene (Li & Dewey, 2011). As for the differential expression analysis, the Empirical analysis of Digital Gene Expression in R was used to analyze the difference among the three groups (Robinson, McCarthy & Smyth, 2009). The expression of genes with a fold change ≥2 or ≤0.5 would be filtered as DEGs. The function analysis of DEGs was carried out by GO analysis and KEGG pathway enrichment analysis using Goatools and KOBAS (Xie et al., 2011).

### Quantitative real-time polymerase chain reaction analysis

In order to confirm the reliability of RNA-seq, qRT-PCR was used to confirm the results. If the results of qRT-PCR were consistent with the ones of RNA-seq, this meant that the reliability of RNA-seq was quite high. We could use the results in the RNA-seq, as a basic database, to explore some potential pathways and mechanisms.

Total RNA (one μg) was reverse-transcribed into cDNA using HiScript^®^ II 1st Strand cDNA Synthesis Kit (Vazyme, Nanjing, China) according to the manufacturer’s protocol, and an amplification mixture was prepared using ChamQ SYBR qPCR Master Mix (Vazyme, Nanjing, China). The mRNA expression was quantified by a real-time fluorescence quantitative PCR system (ABI 7500, Foster City, CA, USA) (Tables S1 and S2) (Johnson et al., 2014). The primers of selected mRNAs (Gereray Biotechnology, China) were used (Table S3). The results were normalized to β-actin expression (Guo et al., 2013). The relative expression of mRNAs was calculated by the method of 2^−ΔΔCt^.

### Statistical analysis

The results were presented as means ± SD. Statistical analysis was carried out by using the software SPSS 22.0. One-way analysis of variance followed by a Student–Newman–Keuls test was used for comparisons of multiple groups. Values of *p* < 0.05 were considered statistically significant.

## Results

### Luteolin attenuated hepatotoxicity caused by METH

**Figure 1:**
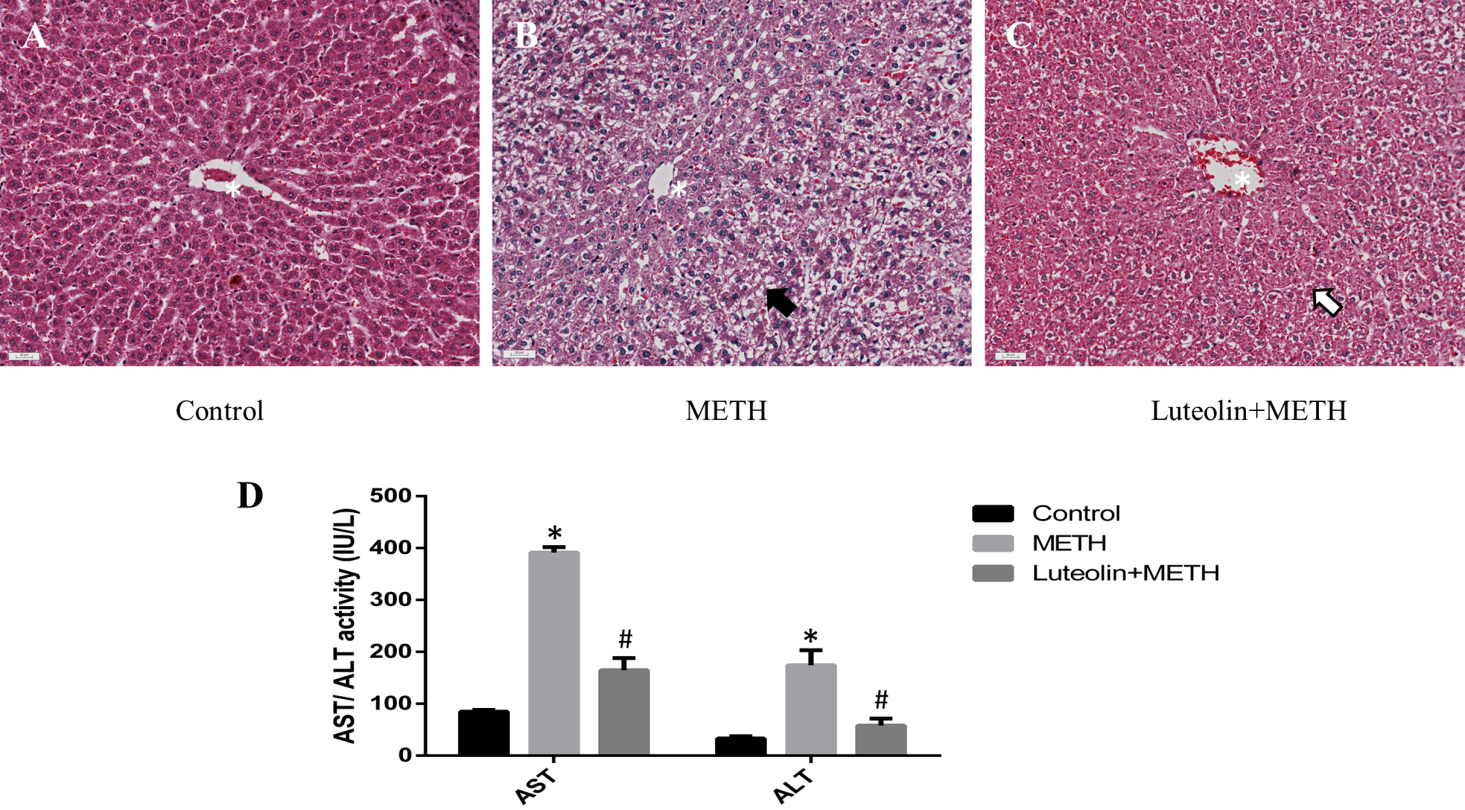
Protective effects of luteolin on liver injury caused by METH were observed by histopathology and biochemical analysis. (A–C) In the METH group, severe hepatocyte ballooning (black arrowhead) caused by METH was observed compared to the control group. In the Luteolin + METH group, the liver injury was obviously attenuated (white arrowhead) compared with the METH group. (D) Compared to the control group, the levels of AST/ALT were significantly increased in the METH group, while in the group pretreated with luteolin, the levels of AST/ALT were obviously decreased 66.67% and 67.16%. *Significantly different from the corresponding control group, *p* < 0.05; ^#^Significantly different from the corresponding METH group, *p* < 0.05.

Histopathological changes could give a direct assessment of the protective effects of luteolin on the hepatotoxicity caused by METH. No obvious injuries were shown in the control group (Fig. 1A). In the METH-treated group, extensive morphological changes could be observed mainly including hepatocyte ballooning (Fig. 1B). In the group pretreated with luteolin, the pathological changes were obviously attenuated compared with the METH group (Fig. 1C).

In order to assess the levels of hepatotoxicity, the levels of serum ALT and AST were determined. The levels of ALT and AST were significantly increased in the group treated with METH compared with the control group. In the group with luteolin pretreatment, the levels of ALT and AST were reduced compared with the METH-treated group (Fig. 1D).

### Differentially expressed genes

**Figure 2:**
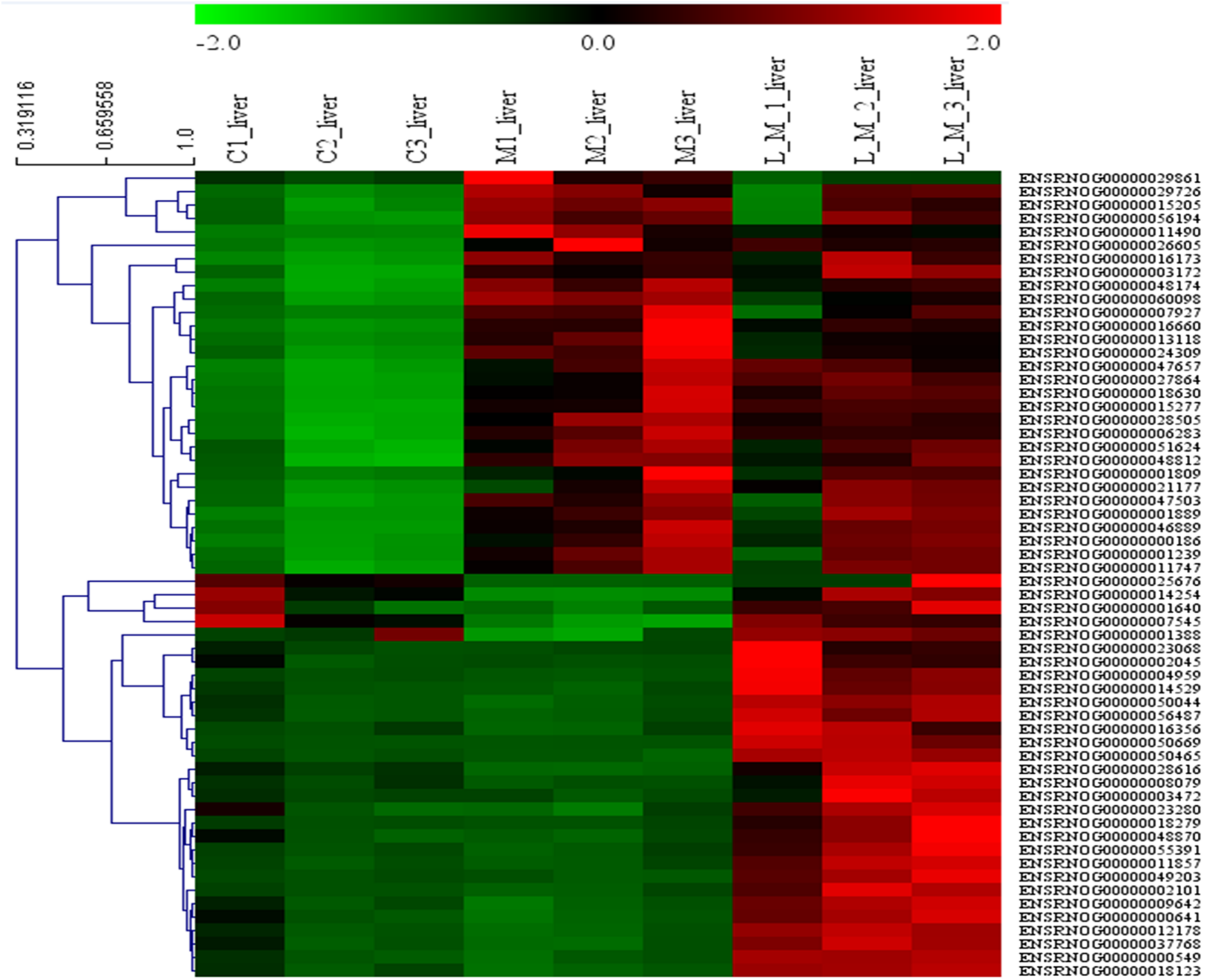
Heatmap of DEGS. Top 60 DEGs, 30 up-regulated and 30 down-regulated DEGs were shown in the heatmap.

**Figure 3:**
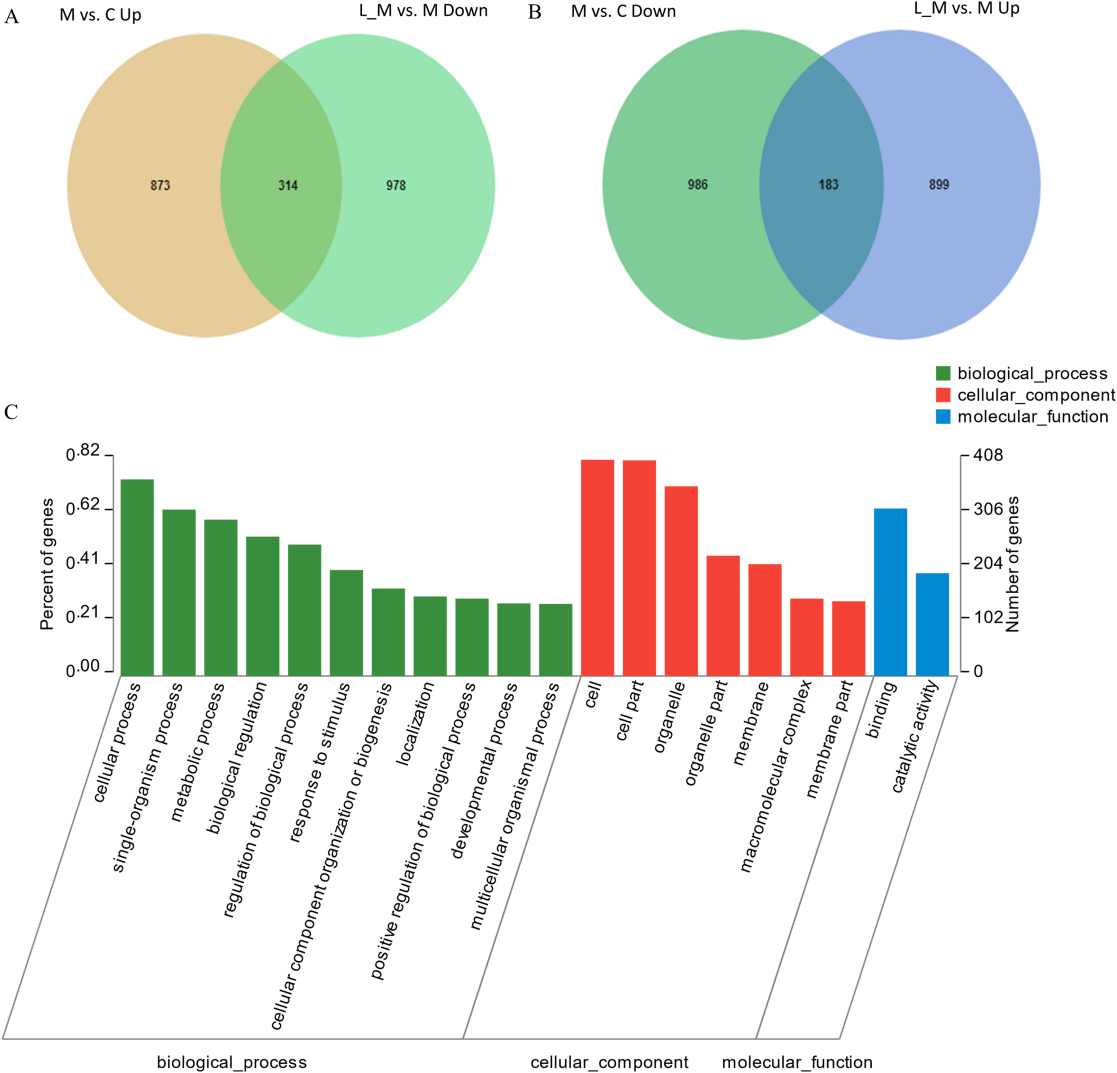
DEGs regulated by Luteolin and GO analysis of the DEGs. (A and B) A total of 497 DEGs were found to have regulatory effects in the METH group by luteolin including 314 up-regulated and 47 down-regulated DEGs in the Venn map. (C) Go analysis of the DEGS.

In order to explore the potential functions of mRNA, we detected DEGs in all the three groups. Compared with the control group, 1,859 DEGs were identified in the METH-treated group including 873 up-regulated and 986 down-regulated DEGs. Meanwhile, in the group pretreated with luteolin, 899 up-regulated and 978 down-regulated DEGs were filtered. Finally, 497 DEGs including 314 up-regulated and 183 down-regulated DEGs which might have protective effects were found. The top 60 DEGs were summarized. A Heatmap and Venn map diagram of the DEGs is shown in Figs. 2, 3A and 3B.

### GO analysis

In order to further predict the biological functions of the selected DEGs, GO analysis was performed to test for cellular components, molecular functions, and biological processes. The 497 DEGs were divided into three groups including cellular components, biological processes, and molecular functions according to the functions and pathways (Fig. 3C).

### KEGG pathway enrichment analysis

Eight pathways were enriched by KEGG pathway analysis (Table 1). Oxidative phosphorylation, Non-alcoholic fatty liver disease (NAFLD), Parkinson’s disease (PD), PPAR signaling pathway, AMPK signaling pathway, Alzheimer’s disease (AD), Metabolism of xenobiotics by cytochrome P450, and Drug metabolism—cytochrome P450, were the enriched pathways.

**Table 1:** Pathway enrichment.

No.	Pathway ID	Pathway	Count
1	map00190	Oxidative phosphorylation	16
2	map05012	Parkinson disease	16
3	map04932	Non-alcoholic fatty liver disease (NAFLD)	14
4	map04152	AMPK signaling pathway	9
5	map00980	Drug metabolism—cytochrome P450	6
6	map05010	Alzheimer disease	16
7	map03320	PPAR signaling pathway	8
8	map00980	Metabolism of xenobiotics by cytochrome P450	6

### Validation of RNA-seq by qRT-PCR

**Figure 4:**
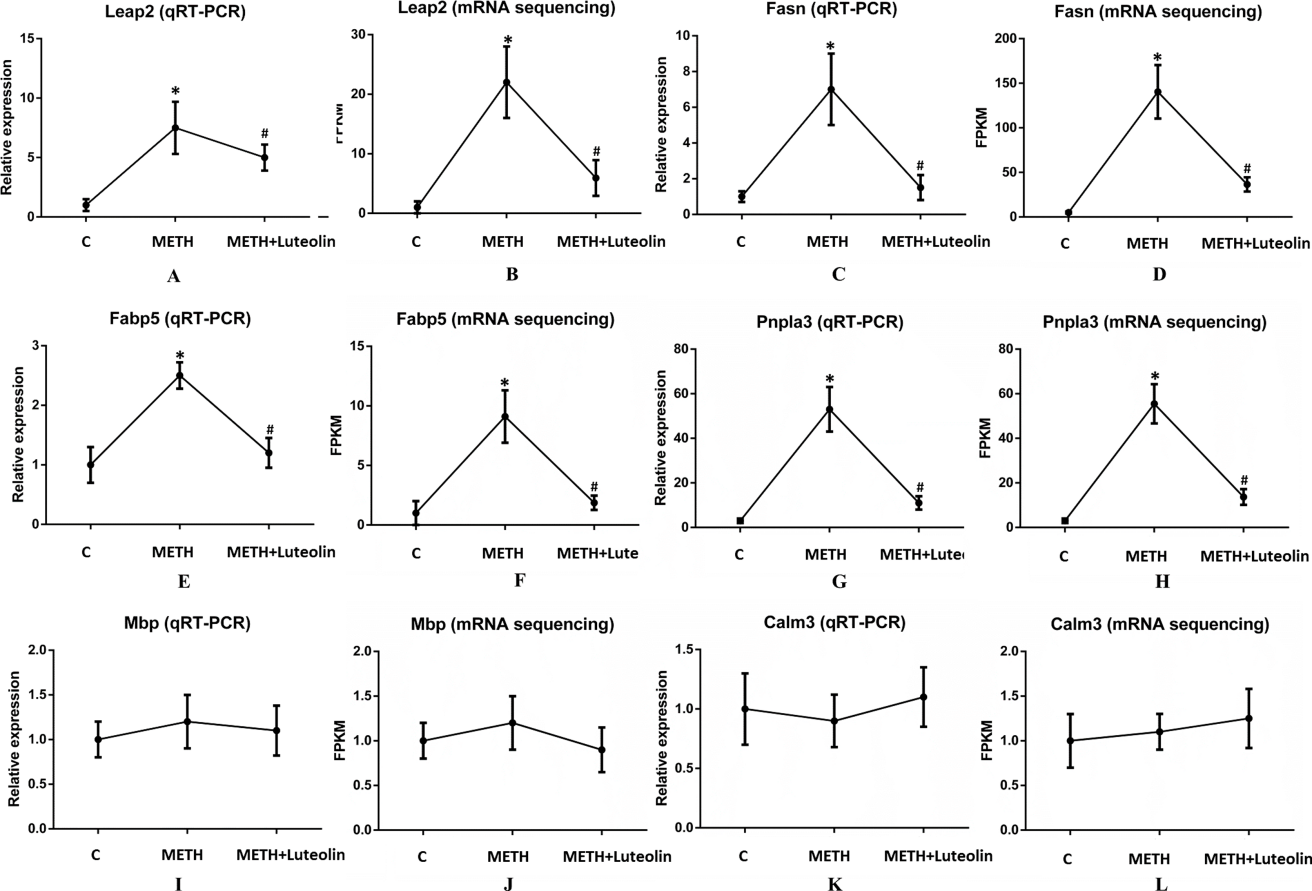
Consistence of results of qRT-PCR and RNA-seq. The results of qRT-PCR were consistent with the ones of RNA-seq (A–L). Leap2 (A, B), Fasn (C, D), Fabp5 (E, F) and Pnpla3 (G, H) were up-regulated DEGs in the METH group compared with the control group, and Mbp (I, J) and Calm3 (K, L) were genes which did not change among the three groups. *Significantly different from the corresponding control group, *p* < 0.05; ^#^Significantly different from the corresponding METH group, *p* < 0.05.

To confirm the accuracy of RNA-seq results, six genes (Leap2, Fasn, Fabp5, Pnpla3, Mbp and Calm3) were randomly selected to verify the results of sequencing using quantitative real-time PCR. Leap2, Fasn, Fabp5 and Pnpla3 were up-regulated DEGs in the METH group compared with the control group, and Mbp and Calm3 were genes which did not change among the three groups. The results of qRT-PCR were consistent with the results of RNA-seq (Fig. 4).

## Discussion

Hepatotoxicity of METH has been confirmed by histopathological examination. Furthermore, the levels of ALT and AST were significantly increased in the group treated with METH compared with the control group. Though the use of only ALT and AST to assess liver injury could be argued as being insufficient since these enzymes are not completely unique to the liver, being found in other tissues, in this study, the ALT/AST elevations were likely from the liver, especially considering the pathology of the liver. In addition, the experimental results of the hepatotoxicity of METH were consistent with those in previous studies (De Carvalho et al., 2018; Halpin, Gunning & Yamamoto, 2013; Wang et al., 2017a; Xie et al., 2018a). In addition, some studies have reported that luteolin has protective effects on the hepatotoxicity of several medicines and toxins including tetrachloromethane (CCl_4_), acetaminophen, mercuric chloride and ethanol (He et al., 2016; Tai et al., 2015; Zhang et al., 2017). Therefore, we hypothesized that luteolin might have protective effects on hepatotoxicity induced by METH. We found that luteolin has a protective effect on hepatotoxicity due to METH using H and E staining and detection of AST/ALT levels. The pathological change of livers in the luteolin–pretreated group was decreased compared with the METH group. And the levels of AST/ALT in the group pretreated with luteolin were also obviously lower than in the METH group. The effect of luteolin on hepatotoxicity caused by METH was confirmed in the animal experiment. To the best of our knowledge, this is the first report that studied the protective effects of luteolin on METH-induced hepatotoxicity. In order to explore the potential mechanism of the protective effect of luteolin on hepatotoxicity, RNA-seq was used to explore the molecular mechanism. Thousands of DEGs were detected after sequencing, but not all detected genes were involved in the mechanism of anti-hepatotoxicity of luteolin. Therefore, an effective strategy for selection of genes should be carried out. Plainly, since METH and luteolin have opposite effects on the liver, the DEGs are regulated by the opposite effect of both METH and luteolin. A total of 497 DEGs including 314 up-regulated and 183 down-regulated DEGs that might have protective effects were found. The subsequent GO analysis and KEGG analysis were both based on these 497 genes.

Given METH causes hepatotoxicity and luteolin can attenuate the hepatotoxicity induced by METH, eight pathways were enriched from the KEGG analysis in present study. Among the eight pathways, Oxidative phosphorylation, NAFLD, PPAR signaling pathway, AMPK signaling pathway, Metabolism of xenobiotics by cytochrome P450, and Drug metabolism—cytochrome P450 were mainly related to hepatotoxicity by METH and the effects of luteolin on hepatotoxicity.

Exposure to METH also altered the expression of the mRNAs involved in the oxidative phosphorylation pathway (Wang et al., 2017b) in which cells use enzymes to oxidize nutrients and then to produce adenosine triphosphate. Some studies have reported that oxidative phosphorylation capacity in the mitochondria would be altered and would influence cellular function (Kim, Chin & Cho, 2017; Xia et al., 2014; Xu et al., 2015). It has been reported that NAFLD is associated with insulin resistance, metabolic syndrome and other metabolic dysfunctions (Jacobs et al., 2011). Interestingly, our group has also found that METH can break the metabolic balance in liver and luteolin could restore the broken metabolic balance caused by METH (unpublished). The AMPK signaling pathway, a vital cellular energy sensor and metabolic regulator, plays a role in cellular energy homeostasis, largely to activate glucose and fatty acid uptake and oxidation (He et al., 2015; Mihaylova & Shaw, 2011). It has been reported that AMPK could reverse drug-induced mitochondria and hepatocyte injury by promoting mitochondria fusion (Kang et al., 2016). The PPAR signaling pathway plays an essential role in the regulation of cellular differentiation and metabolism (Shah, Rader & Millar, 2010). Some studies have reported that the PPAR signaling pathway is involved in liver fibrosis, liver inflammation, and so on (Panebianco et al., 2017; Xia et al., 2014). The signaling pathways of Metabolism of xenobiotics by cytochrome P450, and Drug metabolism—cytochrome P450 are both regulated by cytochrome P450, a kind of enzyme, which could play a role in metabolizing toxins, in the liver especially (Guengerich, 2013). It has been reported that the P450 enzyme could regulate the metabolism of drugs in the liver (Almazroo, Miah & Venkataramanan, 2017; Gao et al., 2017). In fact, NAFLD, PPAR and AMPK signaling pathways are not amongst the recognized mechanisms of METH-induced liver injury. However, because the underlying mechanisms of liver injury caused by METH, as well as the effects of luteolin have not been fully clarified, RNA-seq was carried out to detect DEGs in this study. These selected DEGs were mainly enriched in eight pathways, according to KEGG analysis, including NAFLD, PPAR and AMPK signaling pathways. While speculative, this indicated to use that luteolin might potentially be used for the treatment of liver injury due to METH, although multiple mechanisms are responsible for METH-induced liver injury. Further investigation is needed to clarify the exact effects and mechanisms.

Among the eight enrichment pathways, the two signaling pathways of PD and AD were enriched in the liver tissue treated with METH and luteolin. This phenomenon is interesting, but by reading previous studies we found that METH is closely related to PD and AD (Chen et al., 2019; Kish et al., 2017; Kousik, Carvey & Napier, 2014; Kuehn, 2011; Lappin, Darke & Farrell, 2018; Mursaleen & Stamford, 2016).

Several limitations should be considered in the present study. Because of the design flaws, the luteolin group has not been included. It would be more appropriate to investigate the effect of luteolin on liver injury caused by METH if the luteolin group was included in this study. For using bioinformatics tools to relate RNA-seq data. It is vital to note that changes in mRNAs do not always represent the expressions and functions of proteins. Although a lot of mRNA changes were observed in this study, changes of protein and localization of protein require a more intensive investigation. In addition, while the genes are expressed in opposite directions by METH and luteolin, this does not mean that all of the gene expression induced by METH is harmful and conversely, that all gene expression by luteolin is beneficial. Furthermore, the relevance to humans from rodents, even if the genes are expressed similarly is not guaranteed. Therefore, further investigation is needed.

## Conclusion

In summary, we reported a RNA-seq involved in the effects of luteolin on METH-induced hepatotoxicity and an overview of networks of DEGs upon METH and luteolin exposure. Our research results provided a possible clue concerning the basic pathways involved in and the basic mechanisms of the effects of luteolin in response to METH in vivo.

## Supplemental Information

10.7717/peerj.8529/supp-1 Supplemental Information 1 RT-qPCR conditions.

10.7717/peerj.8529/supp-2 Supplemental Information 2 Thermal cycler parameters.

10.7717/peerj.8529/supp-3 Supplemental Information 3 Gene-specific primer sequences for RT-qPCR.

10.7717/peerj.8529/supp-4 Supplemental Information 4 Raw data of AST and ALT.

## Additional Information and Declarations

### Data Availability

The following information was supplied regarding data availability: The RNA-seq data is available at BioProject: PRJNA529763.
