# Supplementary material for: RNA-sequencing analysis of the effect of luteolin on methamphetamine-induced hepatotoxicity in rats: a preliminary study
Source: PeerJ. 2020 Feb 6;8:e8529. doi: 10.7717/peerj.8529 (PMC7007981; doi:10.7717/peerj.8529)
Supplement: Supplemental Information 1 [file peerj-08-8529-s001.doc]

| Table 1 RT-qPCR conditions |  |
| --- | --- |
| Components | Volume（μl） |
| RNase-free H_2_O | 3 |
| 2×Realtime PCR Mix | 5 |
| Forward primer（10μM） | 0.5 |
| Reverse primer（10μM） | 0.5 |
| cDNA | 1 |
