# Supplementary material for: RNA-sequencing analysis of the effect of luteolin on methamphetamine-induced hepatotoxicity in rats: a preliminary study
Source: PeerJ. 2020 Feb 6;8:e8529. doi: 10.7717/peerj.8529 (PMC7007981; doi:10.7717/peerj.8529)
Supplement: Supplemental Information 2 [file peerj-08-8529-s002.doc]

| Table 2 Thermal cycler parameters |  |  |
| --- | --- | --- |
| Temperature | Time | Cycles |
| 94℃ (Pre degeneration) | 30s | Stage1 1Cycle |
| 95℃ (denature) | 5s | Stage2 40Cycles |
| 61℃ (Primer annealing) | 30s |
| 72℃ (extension) | 30s |
| 95℃ | 15s | Melting Curve 1Cycle |
| 55℃ | 15s |
| 95℃ | 15s |
