# Supplementary material for: RNA-sequencing analysis of the effect of luteolin on methamphetamine-induced hepatotoxicity in rats: a preliminary study
Source: PeerJ. 2020 Feb 6;8:e8529. doi: 10.7717/peerj.8529 (PMC7007981; doi:10.7717/peerj.8529)
Supplement: Supplemental Information 3 [file peerj-08-8529-s003.doc]

| Table 3 Gene-specific primer sequences for RT-qPCR | | |
| --- | --- | --- |
| Gene | Primer | Sequence (5´-3´) |
| Rattus norvegicus (Norway rat) |  |  |
| β-actin | Foward | CCCATCTATGAGGGTTACGC |
|  | Reverse | TTTAATGTCACGCACGATTTC |
| Leap2 | Foward | CAGCTAAAACTCTTTGCAGTGC |
|  | Reverse | TCTCCGGGATCTCTTTGCTGA |
| Fasn | Foward | GGAGGTGGTGATAGCCGGTAT |
|  | Reverse | TGGGTAATCCATAGAGCCCAG |
| Fabp5 | Foward | TGAAAGAGCTAGGAGTAGGACTG |
|  | Reverse | CTCTCGGTTTTGACCGTGATG |
| Pnpla3 | Foward | GCGGCTTCCTAGGCTTCTAC |
|  | Reverse | CCATGATGTGATCGAGAGGGA |
| Mbp | Foward | GGCGGTGACAGACTCCAAG |
|  | Reverse | GAAGCTCGTCGGACTCTGAG |
| Calm3 | Foward | GATGGCACCATTACCACCAAG |
|  | Reverse | CGCTGTCTGTATCCTTCATCTTT |
